# Preparation and *In-vitro* Evaluation of Controlled Release PLGA Microparticles Containing Triptoreline

**Published:** 2010

**Authors:** Alireza Mahboubian, Seyyed Kazem Hashemein, Shadi Moghadam, Fatemeh Atyabi, Rassoul Dinarvand

**Affiliations:** a *Faculty of Pharmacy, Tehran University of Medical Sciences, Tehran, Iran.*; b * Medical Nanotechnology Research Centre, Tehran University of Medical Sciences, Tehran, Iran.*

**Keywords:** Microsphere, Triptoreline, Double emulsion, Controlled-release, PLGA

## Abstract

Triptoreline is a potent agonist of luteinizing hormone-releasing hormone, currently used in the treatment of prostatic cancer where therapy may be required over months or years. Frequent injection of drug decreases patients’ compliance. The present study describes the formulation of a sustained release microparticulate drug delivery system containing triptoreline acetate, using poly (D,L lactide-co-glycolide) (PLGA). Biodegradable microspheres were prepared using 50 : 50 PLGA by a water in-oil-in-water (w/o/w) double emulsion-solvent evaporation procedure and characterized for drug content and drug release rate using the a HPLC method, particle size distribution using the laser diffraction method, and surface morphology using scanning electron microscopy and drug release rate. Effect of critical process parameters and formulation variables; *i.e*. volume of inner water phase, addition of NaCl to the outer aqueous phase (W2), addition of different types and amounts of emulsifying agents on microsphere characteristics; were investigated. Microspheres prepared were spherical with a smooth surface, but addition of poloxamer to the first emulsion produced microspheres with large pores. Size of microparticles was dependent on the type, as well as the amount of co-encapsulated surfactants. Increasing the inner water phase volume resulted in larger particles with a lower encapsulation efficiency. Low concentrations of Span 20 decreased triptoreline release rate, whereas the addition of poloxamer or high concentrations of Span 20 increased the drug release rateit. In conclusion, by selecting an appropriate level of the investigated parameters, spherical microparticles with encapsulation efficiencies higher than 90% and a prolonged triptoreline release over 45 days were obtained.

## Introduction

Proteins are an integral part of the body as they carry out all the important physiological and biological processes, like ligands for signaling, enzymes for biotransformation reactions, receptors for pharmacological response elucidation, antibodies in immune system interactions, transcription, and translation (1). Over the last 20 years, a large number of recombinant proteins have been investigated to find their therapeutic applications and many of them have been formulated as drugs, forming a new class of therapeutic agents. Ailments that can be treated effectively by this new class of therapeutic agents include cancers, autoimmune diseases, memory impairments, mental disorders, hypertension and certain cardiovascular and metabolic diseases (2).

The use of these protein drugs is limited clinically, since proteins have unique requirements and limitations for delivery compared with low molecular weight molecules. Generally, they have short plasma half-lives, are incapable of diffusing through biological membranes, and are not stable in the gastrointestinal tract (3), which makes oral bioavailability low. Many proteins currently being developed are aimed at chronic conditions where therapy may be required over months or years. Alternative administration by frequent injections to keep the protein drug at effective concentrations is tedious, expensive, and has poor patient compliance. Therefore, development of sustained release injectable dosage forms becomes necessary to improve the efficacy of peptide drugs and eliminate the need for frequent administration (4-8). 

Biodegradable microspheres were shown to improve the bioavailability of peptides by protecting them from physical degradation and proteolysis in body fluids. Poly(D,L-lactide) (PLA) and poly (D,L-lactide-co-glycolide) (PLGA) are the most widely used and well-characterized materials for the preparation of biodegradable microspheres (9-11). 

Triptoreline, the peptide drug used in this study, is a potent agonist of luteinizing hormone-releasing hormone (LHRH) currently used for the treatment of prostate cancer, endometriosis and precocious puberty (12-13). 

In the present study, controlled release PLGA microspheres containing triptoreline were prepared. A w/o/w solvent evaporation technique was selected for microsphere preparation, as this is the most commonly used method to encapsulate hydrophilic drugs, especially protein and peptide drugs, into polymeric microspheres (14-16). Two important prerequisites for high encapsulation efficiencies by the w/o/w method are: (a) the insolubility of the drug in the organic polymer solution, which separates the internal phase from the external aqueous phase, and (b) the fine dispersion of the aqueous drug solution into the organic polymer solution to form a w/o emulsion (17). Size and release properties of microspheres are the key considerations to design microsphere delivery systems. Since the release kinetics of protein dominantly depends on polymer nature, morphology and particle size, fundamental understanding of the relationship among these key characteristics and release mechanisms is essential to yield useful products (18, 19).

In this work, a biodegradable polyester (PLGA 50 : 50) was used to encapsulate triptoreline acetate within sustained release microspheres. The aim of this study is to further investigate the effect of fabrication variables on morphology, size distribution, drug encapsulation efficiency, and release kinetics profiles at 37°C, which is close to human body temperature, and to study the relationship among these factors. 

## Experimental

*Materials*

Triptoreline acetate, [5-Oxopro-His-Trp-Ser-Tyr-O-Trp-Leu-Arg-Pro-GlyNH2] was obtained from Sinopep (China). Poly (D, L-Lactic-co-glycolic acid) 50:50 (PLGA 50 : 50) Resomer^®^RG 504H was supplied by Boehringer-Ingelheim (Germany). Poly (vinyl alcohol) (PVA) (MW 22000, 88% hydrolyzed) was supplied by Acros (USA). Poloxamer 407 was purchased from Synopharm (Germany). Dichloromethane, Tween 80, Span 20 and sodium azide were obtained from Merck (Germany). 

*Preparation of microspheres*

Triptoreline acetate-loaded microspheres were prepared by a double emulsion-solvent evaporation technique. Briefly, 500 mg PLGA was dissolved in 5 mL dichloromethane (oil phase). An aqueous solution containing different amounts of triptoreline acetate was prepared separately (inner aqueous phase or W_1_).

The first aqueous phase (w_1_) was emulsified into the oil phase (containing PLGA), using a high-speed homogenizer (T18 basic, IKA, Germany) at different speeds and time durations. Afterwards, the primary emulsion was added to 250 mL of 0.5% w/v poly-vinyl alcohol solution, while stirring with a mechanical mixer at different rates to form the w_1_/o/w_2_ double emulsion. Mixing was continued for 8 h at room temperature, until complete evaporation of dichloromethane. The resulted microparticles were collected by filtration, washed with water and dried overnight at room temperature. In some formulations, 2% w/v poloxamer 407, 1% or 10% v/v Span 20 were added to the first (w_1_/o) emulsion. In some formulations, 5% w/v sodium chloride was added to the external water phase (w_2_) in the presence or absence of emulsifier.

*Scanning electron microscopy (SEM)*

The morphology of microparticles was examined by scanning electron microscopy (MW2300, Cam Scan, England). Samples were mounted on metal stubs and sputter-coated with gold for 4 min, prior to examination. 

*Particle size determination*

The mean diameter of microspheres was determined by laser diffractometer (Mastersizer X, Malvern Instrument, UK). Microparticles were suspended in a 0.3% aqueous solution of Tween 80 and sonicated for 15 s prior to particle size determination.

*Encapsulation efficiency*

A known amount of triptoreline-loaded microspheres was added to 2 mL dichloromethane to dissolve PLGA. Phosphate buffer pH 5 (2 mL) was added into the previous solution and agitated for 10 min to extract the peptide. The amount of encapsulated triptoreline acetate was assayed in the aqueous phase by HPLC. The HPLC system consisted of a pump (K l00l, Knauer, Germany), and a UV detector (K2600, Knauer, Germany) and a Perfectsil target ODS-3, 5μm, 150×4.6 mm column (MZ-Analysentechnik, Spain). The mobile phase consisted of 30% v/v acetonitrile: Phosphate buffer pH 6.5 (70 : 30) and detection was carried out at 220 nm. The injection volume was 20 μL and the flow rate was 1.0 mL/min.

The encapsulation efficiency was calculated as follows:

Encapsulation efficiency (%) = [actual drug loading /theoretical drug loading] × 100

*In-vitro drug release study*

For this purpose, 100 mg of drug-loaded microspheres were added to the test tube containing 7 mL 0.05 M phosphate buffer solution pH 7.4 alongside 0.02% Tween 80 and 0.02% sodium azide and suspended thoroughly. The tube was placed in a 37°C shaker water bath at 130 strokes/min. At different time intervals, the tube was centrifuged at 12000 rpm for 10 min and 5 mL of the supernatant layer was removed for analysis and an equal volume of fresh phosphate buffer was added to the test tube. The amount of released triptoreline was assayed, using a HPLC method, as described in the encapsulation efficiency study.

*Statistical analysis*

One-way analysis of variance (ANOVA) was performed on the data to assess the impact of the formulation variables on the results. P-values less than 0.05 were considered as significant. All the calculations were performed, using a statistical software program (SPSS^®^ 11.5, Microsoft).

## Results and Discussion

**Figure 1:**
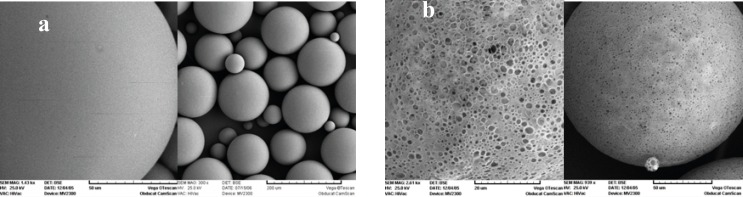
Scanning electron micrographs of PLGA microspheres prepared: (a) with NaCl, and (b) without 5% NaCl in the external aqueous phase

Figure 1 shows the SEM photographs of the microspheres prepared, with or without 5% NaCl in the outer water phase (w_2_). Both formulations were spherical, but addition of NaCl resulted in a smoother surface with a lower porosity. The presence of pores on the surface of microspheres, in the absence of NaCl, could be attributed to the migration of w_2 _towards w_1 _due to the lower osmotic pressure of w_2_. As can be seen in Table 1, presence of NaCl decreased the mean particle size significantly (105 μm*. *159 μm).

**Table 1:** The effect of different formulation parameters on the particle size and triptoreline encapsulation efficiency of PLGA microspheres

**Formulation number**	**W**_1_ **volume (mL)**	**NaCl in w**_2_	**Surfactant used**	**Particle size (μm)**	**Encapsulation efficiency (%)**
1	0.5	-	-	159 ± 5.71	91.7 ± 0.54
2	1.0	-	-	183 ± 3.47	26.2 ± 0.32
3	0.5	+	-	105 ± 6.80	92.3 ± 0.14
4	0.5	+	1% Span 20	82 ± 4.95	41.1 ± 0.52
5	0.5	+	10% Span 20	21 ± 5.20	6.1 ± 0.61
6	0.5	+	2% Poloxamer	130 ± 3.50	85.6 ± 0.30

 Increasing the osmotic pressure of w_2_ leads to water migration from w_1_ to w_2_ and a rapid shrinkage of the droplets. This phenomenon results in smaller microparticles (20). It was reported previously that the shrunk and dense surface acts as a barrier against losing drug during the microencapsulation process and improves drug loading (9). But in the present study, there was no significant difference between encapsulation efficiency of formulations prepared with or without NaCl (92% *vs. *91%). This could be due to electric interactions between negatively charged Carboxyl ions in PLGA and positively charged ammonium ions in triptoreline, resulting in the formation of a barrier which efficiently prevents triptoreline leaving or diffusing from the microspheres during the preparation process. This trend was similar to the results obtained by Cui *et al*, for the encapsulation of melittin (21). 

**Figure 2:**
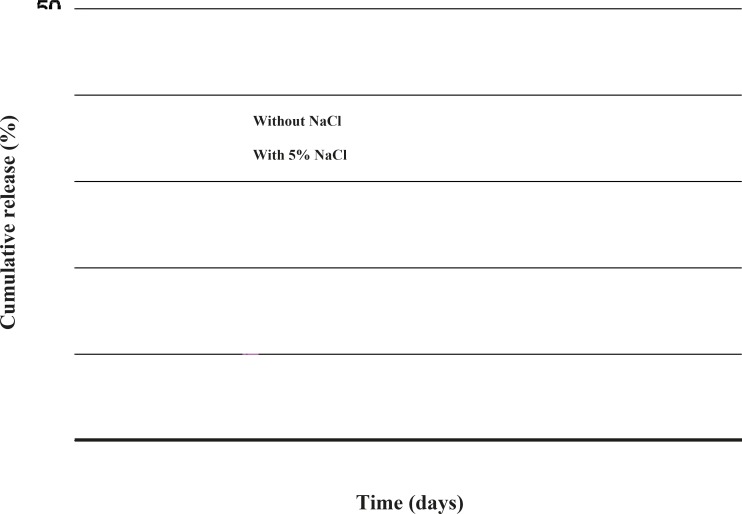
The effect of presence of NaCl in the outer water phase during microsphere preparation on triptoreline release rate (n = 3; mean ± standard deviation).

Effect of NaCl on triptoreline release is presented in Figure 2. The burst release was decreased from 11 to 2% during the first 24 h, as a result of the presence of NaCl in w_2_. This trend was continued until the end of first week. In the absence of NaCl, osmotic gradient between w_1_ and w_2_ forces water migration towards w_2_ which would take some of the drug molecules to the surface of microparticles. These surface drugs liberate rapidly upon contact with the dissolution medium (22). 

**Figure 3:**
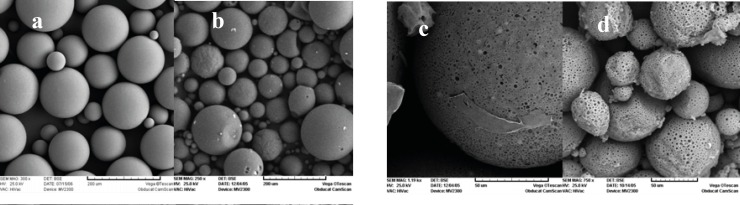
Scanning electron micrographs of triptoreline microspheres prepared with different inner water phase volumes: (a) w_1_ = 0.3 mL, (b) w_1_ = 0.5 mL, (c) w_1_ = 1 mL, and (d) w_1_ = 2 mL

Increasing the internal aqueous phase volume from 0.3 to 2 mL, increased the porosity of matrix and in the case of 2 mL of w_1_, microspheres were not spherical any more (Figure 3). Some researchers have demonstrated that the volume of the internal aqueous phase influences the microstructure (porosity) of the microspheres (23, 24). 

Table 1 shows a direct relation between w_1_ volume and microsphere size. Decreasing the w_1_ volume from 1 to 0.5 mL, reduced the mean particle size from 183 to 159 μm. Apparently, addition of w_1_ volume provided a greater resistance to mechanical break-down during the second emulsification process. This finding is consistent with the fact that emulsion viscosity increases as the internal aqueous phase volume fraction increases (25, 26). 

The encapsulation efficiency results have been shown in Table 1. When the inner water phase volume decreased from 1 to 0.5 mL, the drug encapsulation efficiency increased drastically (from 26.2% to 91.7%). As scanning electron micrographs revealed, the more porous structure of microparticles prepared with a higher volume of w_1_ facilitates the migration of drug molecules from w_1_ to w_2_ during the microencapsulation process, leading to lower encapsulation efficiencies. 

**Figure 4:**
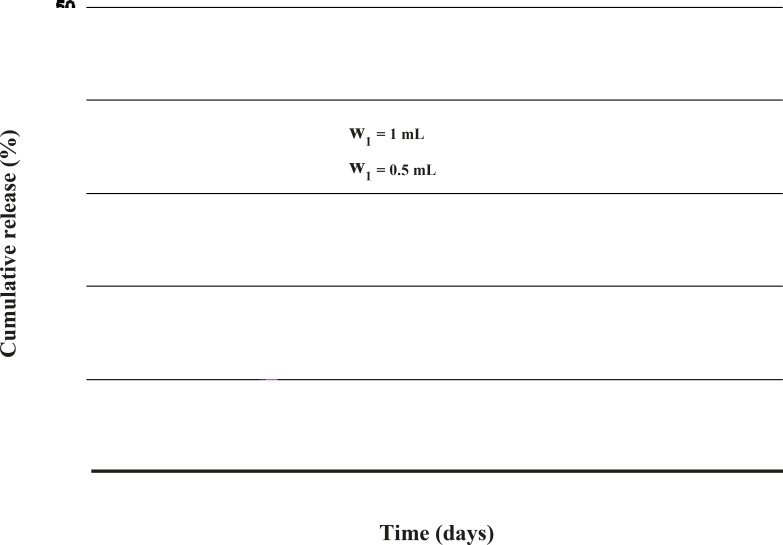
Effect of the inner water phase volume on triptoreline release from PLGA microspheres (n = 3, mean ± standard deviation).

In contrary to expectation, triptoreline release rate from microspheres prepared by a larger volume of w_1_ was slower than formulation with a denser structure (Figure 4). This could be attributed to the higher encapsulation efficiency of the latter formulation, which provides a greater concentration gradient between the microsphere matrix and the release medium. 

**Figure 5:**
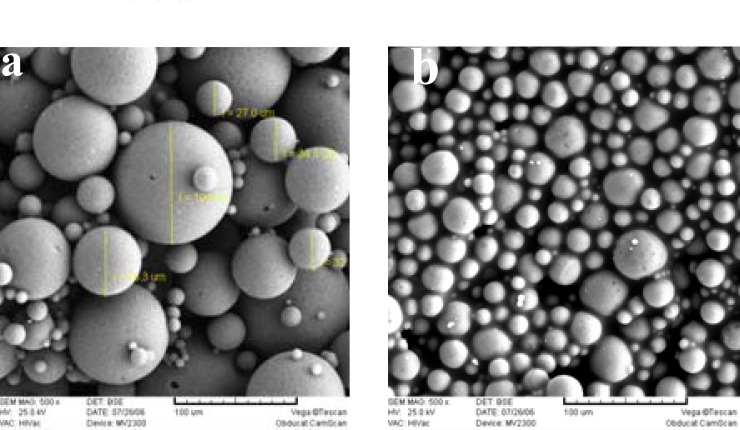
Scanning electron micrographs of triptoreline microspheres prepared with different amounts of Span 20: (a) 1% v/v, and (b) 10% v/v

The effect of Span 20, a non-ionic surfactant, added to the first emulsion during microsphere preparation process, on morphology, average particle size, protein encapsulation and drug release rate was examined. Figure 5 shows that microspheres prepared with either 1 or 10% v/v Span 20 were spherical, but with different particle size. By increasing the amount of emulsifier from 1 to 10%, the mean diameter of microspheres decreased from 82 μm to 21 μm (1). This size reduction may be attributed to the presence of surfactant molecules at the o/w_2_ interface, which facilitates the formation of smaller emulsion droplets, thereby reduces the size of the final microspheres (27). In general, droplet size is directly proportional to the interfacial tension between the dispersed and continuous phase of emulsion. Thus, any decrease in the interfacial tension in the presence of emulsifiers gives rise to a reduction in the microsphere size (28).

Co-encapsulation of Span 20 led to a decrease in triptoreline encapsulation efficiency, which was proportional to the ratio of surfactant (1). 

Aqueous solubility of triptoreline in the presence of Span 20 is increased and therefore it has a greater chance to escape from emulsion droplets to the outer phase. 

**Figure 6:**
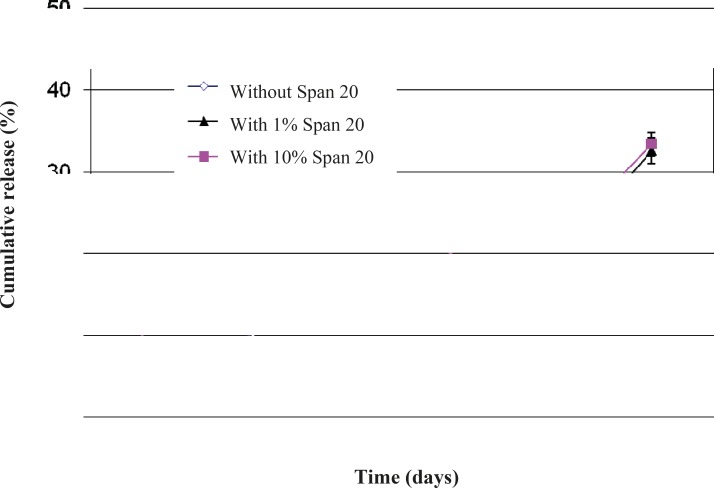
Effect of co-encapsulation of Span 20 on triptoreline release from PLGA microspheres (n = 3, mean ± standard deviation).

Figure 6 presents the effect of Span 20 on triptoreline release from microspheres. During the first week, microspheres without Span released 4% of their drug content, while microspheres containing 1 or 10% Span released 1 and 14% of their drug content, respectively. Addition of 1% v/v Span 20 decreased the drug release rate. This behavior is probably the result of improving the w_1_/o emulsion stability and therefore a better drug dispersion in the polymeric matrix (29) and a lower amount of drug molecules close to the surface of microparticles. In contrast, a higher amount of Span 20 (10%) led to a faster drug release rate by increasing the hydrophilic channels inside the hydrophobic PLGA matrix. 

**Figure 7:**
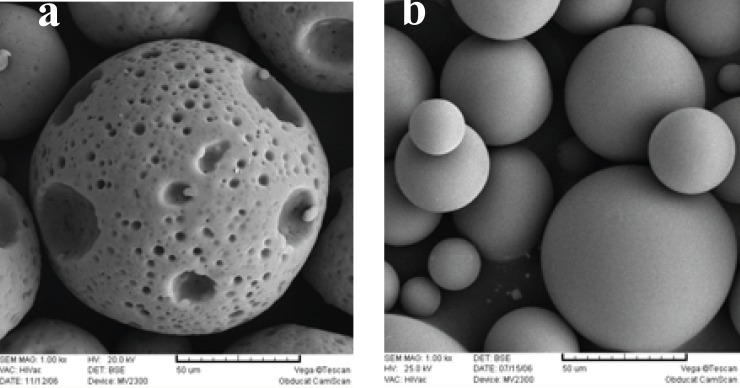
Scanning electron micrographs of triptoreline microspheres prepared: (a) with 2% Poloxamer 407 and (b) without Poloxamer 407

Figure 7 shows the morphological change in PLGA microspheres, from a non-porous to a porous structure, by addition of 2% w/v Poloxamer 407. These porous microspheres had a larger particle size, compared to the microspheres prepared without Poloxamer 407 (1). This was caused by an increase in the fractional volume of the hydrophilic pluronic phase within the emulsion droplets, which in turn imbibed more water and hence enlarging them in size (20). Poloxamer 407 decreased triptoreline encapsulation efficiency (a 7% decrease). It has already been reported that, in the absence of a stabilizer, an interfacial film is formed by the interaction between the protein and the polymer which stabilizes the micro-droplets of the w_1_/o primary emulsion. However, the incorporation of Poloxamer 407 hinders the formation of the stabilizing film because of the competition phenomenon between the Poloxamer and the protein in their interaction with the polymer. This phenomenon leads to the decrease in encapsulation efficiency (30). 

**Figure 8:**
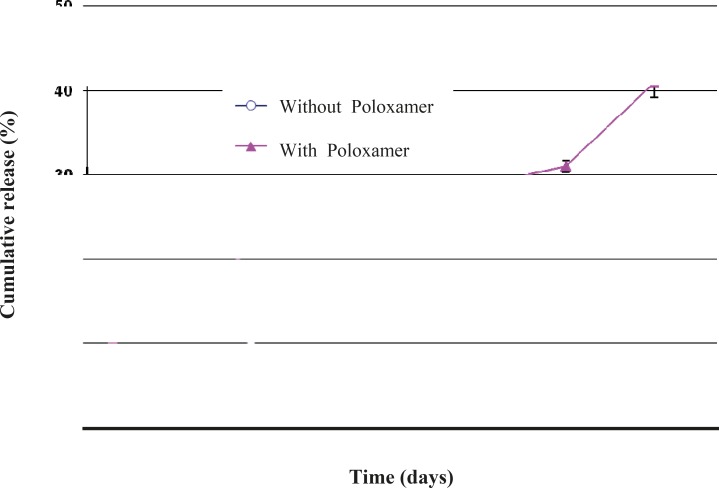
Effect of co-encapsulation of 2% Poloxamer 407 on triptoreline release from PLGA microspheres (n = 3, mean ± standard deviation).

Another interesting observation was that the co-encapsulation of Poloxamer 407 led to a faster triptoreline release from the microspheres prepared by the w/o/w solvent evaporation technique (Figure 8). This result could be explained by the reduced triptoreline – PLGA interaction caused by the presence of Poloxamer 407, as indicated above. Also, the porous structure of these microspheres provides the possibility for triptoreline molecules to rapidly diffuse out through the water-filled pores and inter-connected channels. 

In conclusion, the uniform-sized biodegradable PLGA microspheres containing triptoreline were successfully prepared by double emulsion solvent evaporation method. Various factors related to the preparation process, influenced the drug encapsulation efficiency and the cumulative drug release was subsequently investigated. The results indicated that the drug encapsulation efficiency and the cumulative drug release rates were affected by the presence of NaCl in the outer water phase, inner water phase volume, type and concentration of co-encapsulated surfactant. Ultimately, spherical PLGA microparticles with encapsulation efficiencies higher than 90% and prolonged triptoreline release over 45 days were obtained.

## References

[1] Sinha VR, Trehan A (2003). Biodegradable microspheres for protein delivery. J. Control. Rel.

[2] Banga AK, Chien YW (1988). Systemic delivery of therapeutic peptides and proteins. Int. J. Pharm.

[3] Sanders LM (1990). Drug delivery systems and routes of administration of peptide and protein drugs. Eur. J. Drug Metab. Pharmacokinet.

[4] Mehta RC, Thanoo BC, DeLuca PP (1996). Peptide containing microspheres from low molecular weight and hydrophilic poly(d,b-lactide-co-glycolide). J. Control. Rel.

[5] Chiba M, Hanes J, Langer R (1997). Controlled protein delivery from biodegradable tyrosine-containing poly (anhydride-co-imide) microspheres. Biomaterials.

[6] Ravivarapu HB, Burton K, DeLuca PP (2000). Polymer and microsphere blending to alter the release of a peptide from PLGA microspheres. Eur. J. Pharm. Biopharm.

[7] Hausberger AG, DeLuca PP (1995). Characterization of biodegradable poly (D,L-lactide-co-glycolide) polymers and microspeheres. J. Pharm. Biomed. Anal.

[8] Kostanski JW, DeLuca PP (2000). A novel in-vitro release technique for peptide-containing biodegradable microspheres. AAPS PharmSciTech.

[9] Dinarvand R, Moghadam HS, Sheikhi A, Atyabi F (2005). Effect of surfactant HLB and different formulation variables on the properties of poly-D,L-lactide microspheres of naltrexone prepared by double emulsion technique. J. Microencapsul.

[10] Orafai H, Kallinteri P, Garnett M, Huggins S, Hutcheon G, Pourcain C (2008). Novel poly (glycerol-adipate) polymers used for nanoparticle making: Astudy of surface free energy. Iranian J. Pharm. Res.

[11] Alaee M, Moghadam SH, Sayyar P, Atyabi F, Dinarvand R (2009). Preparation of a reservoir type levonorgestrel delivery system using high molecular weight poly L-lactide. Iranian J. Pharm Res.

[12] Trachtenberg J (1983). The treatment of metastatic prostatic cancer with a potent luteinizing hormone releasing hormone analogue. J. Urol.

[13] Wojciechowski NJ, Carter CA, Skoutakis VA (1986). Leuprolide: A gonadotropin-releasing hormone analog for the palliative treatment of prostatic cancer. Drug Intell. Clin. Pharm.

[14] Ogawa Y, Yamamoto M, Okada H, Yashiki T, Shimamoto T (1988). A new technique to efficiently entrap leuprolide acetate into microcapsules of polylactic acid or copoly(lactic/glycolic) acid. Chem. Pharm. Bull.

[15] Boury F, Marchais H, Proust EJ, Benoit PJ (1997). Bovine serum albumin release from poly (α-hydroxy acid) microspheres: Effects of polymer molecular weight and surface properties. J. Control. Rel.

[16] Alonso JM, Gupta KR, Min C, Siber RG, Langer R (1994). Biodegradable microspheres as controlled-release tetanus toxoid delivery systems. Vaccine.

[17] Alex R, Bodmeier R (1990). Encapsulation of water-soluble drugs by a modified solvent evaporation method. I. Effect of process and formulation variables on drug entrapment. J. Microencapsul.

[18] Shah SS, Cha Y, Pitt GC (1992). Poly (glycolic acid-co-DL-lactic acid): Diffusion or degradation controlled drug delivery? J. Control. Rel.

[19] Langer R, Siegel R, Brown L, Leong K, Kost J, Edelman E (1986). Controlled release: three mechanisms. Chemtech.

[20] Kim KH, Chung JH, Park GT (2006). Biodegradable polymeric microspheres with “open/closed” pores for sustained release of human growth hormone. J. Control. Rel.

[21] Cui F, Cun D, Tao A, Yang M, Shi K, Zhao M, Guan Y (2005). Preparation and characterization of melittin-loaded poly (DL-lactic acid) or poly (DL-lactic-co-glycolic acid) microspheres made by the double emulsion method. J. Control. Rel.

[22] Liu R, Huang SS, Wan HY, Ma HG, Su GZ (2006). Preparation of insulin-loaded PLA/PLGA microcapsules by a novel membrane emulsification method and its release in-vitro. Colloids and Surfaces B: Biointerfaces.

[23] Kissel T, Li XY, Volland C, Gorich S, Koneberg R (1996). Parenteral protein delivery systems using biodegradable polyesters of ABA block structure, containing hydrophobic poly(lactide-co-glycolide) A blocks and hydrophilic poly(ethylene oxide) B blocks. J. Control. Rel.

[24] Du L, Cheng J, Chi Q, Qie J, Liu Y, Mei X (2006). Biodegradable PLGA microspheres as a sustained release system for a new Luteinizing Hormone-Releasing Hormone (LHRH) antagonist. Chem. Pharm. Bull.

[25] Crotts G, Park TG (1995). Preparation of porous and nonporous biodegradable polymeric hollow microspheres. J. Control. Rel.

[26] Jeffery H, Davis SS, O’Hagan DT (1993). The preparation and characterization of poly (lactide-co-glycolide) microparticles. II. The entrapment of a model protein using a (water-in-oil)-in-water emulsion solvent evaporation technique. Pharm. Res.

[27] Rosa GD, Iommelli R, La Rotonda MI, Miro A, Quaglia F (2000). Influence of the co-encapsulation of different non-ionic surfactants on the properties of PLGA insulin-loaded microspheres. J. Control. Rel.

[28] Sah S (1997). Microencapsulation techniques using ethyl acetate as a dispersed solvent: Effects of its extraction rate on the characteristics of PLGA microspheres. J. Control. Rel.

[29] Perugini P, Genta I, Conti B, Modena T, Pavanetto F (2001). Long-term release of clodronate from biodegradable microspheres. AAPS PharmSciTech.

[30] Blanco D, Alonso MJ (1998). Protein encapsulation and release from poly (lactide-co-glycolide) microspheres: Effect of the protein and polymer properties and of the co- encapsulation of surfactants. Eur. J. Pharm. Biopharm.

